# The effectiveness of the early orthodontic correction of functional unilateral posterior crossbite in the mixed dentition period: a systematic review and meta-analysis

**DOI:** 10.1186/s40510-022-00398-4

**Published:** 2022-02-14

**Authors:** Danya Hassan Alsawaf, Salam Ghazwan Almaasarani, Mohammad Y. Hajeer, Nada Rajeh

**Affiliations:** Department of Orthodontics, University of Damascus Dental School, Damascus, Syria

**Keywords:** Crossbite, Functional shift, Interceptive orthodontics, Mixed dentition, Meta-analysis

## Abstract

**Objective:**

This systematic review and meta-analysis aimed to critically appraise the available evidence of the effectiveness of early intervention of functional unilateral posterior crossbites (FPXB) between the ages of 6 and 12 years.

**Materials and methods:**

Electronic search in four databases (PubMed, Scopus, Embase, and Google Scholar) for randomized controlled trials (RCTs) and controlled clinical trials (CCTs) was performed between 1^st^ January 1990 and 31^st^ October 2021. Methodological index for non-randomized studies (MINORS) for CCTs and Cochrane's risk of bias tool for RCTs were applied. The certainty of the evidence was evaluated according to the grading of recommendations, assessment, development, and evaluation (GRADE) approach**.**

**Results:**

Nine studies (6 RCTs and 3 CCTs) were included in this review, and six of them were appropriate for quantitative synthesis. The meta-analysis revealed that the quad-helix (QH) was more effective than expansion plates (EP) in increasing the intermolar width (WMD = 1.25; 95% CI 0.75, 1.75; *P* < 0.001), and decreasing treatment time (WMD = − 3.36; 95% CI − 4.97, − 1.75; *P* < 0.001). The relapse rate at 5.6 years post-treatment was greater in the QH group than in the EP group (RR = 3.00); however, the difference was statistically insignificant. There was no significant difference between the QH and the EP in other outcome measures. When assessing the rapid maxillary expansion (RME), only one RCT compared the RME with an untreated control group and reported a significant increase in the maxillary intermolar and intercanine width (*P* < 0.001, *P* = 0.002, respectively) and a significant decrease in lower midline deviation (*P* < 0.001)**.**

**Conclusion:**

There is weak to moderate evidence that the treatment of functional posterior crossbite (FPXB) by the QH increased the maxillary intermolar width and the success rate and decreased the treatment duration compared to the EP. The relapse percentage was greater in the QH group. There is very weak evidence that the mandibular midline correction rate did not differ significantly between the QH and the EP modalities. The RME using the Hyrax appliance corrected the FPXB successfully; however, the strength of evidence in this regard is very low. As the quality of evidence ranged from very low to moderate in this review, we confirm the need for more RCTs with different expansion appliances in the early treatment of FPXB.

**Supplementary Information:**

The online version contains supplementary material available at 10.1186/s40510-022-00398-4.

## Background

Posterior crossbite is a malocclusion seen frequently in the deciduous and mixed dentitions (8% and 22%, respectively) [1, 2]. It can be unilateral or bilateral and might develop during the mixed dentition [3–5]. The etiology of the crossbite might be dental, skeletal, or functional alone, or in combination [6]. Factors involved in the etiology of the crossbite, besides heredity, are sucking habits [7] and impaired nasal breathing caused by, for example, enlarged tonsils and adenoids [8].

The most common form of posterior crossbites is the unilateral one with a functional shift of the mandible toward the crossbite side. Unilateral posterior crossbite with functional mandibular shift occurs as a sequelae of constricted maxillary arch, which is usually seen in children between 3 and 12 years of age [9]. Its prevalence has been documented to range from 80 to 97% of posterior crossbites in mixed dentition [4, 10]. In children suffering from a functional unilateral posterior crossbite (FPXB), the maxillary complex is often constricted [1, 3]. Because of this transverse maxillary deficiency, frequently more crowding is seen in the maxilla than in the mandible. The crossbite side in a FPXB patient often shows a partial or full Class II molar relationship; the non-crossbite side shows a Class I relationship due to rotational closure of the mandible [11].

Some reports suggest that posterior crossbite might increase the risk of later temporomandibular joint dysfunction [12]; however, other studies found this association weak and conflicting [12, 13]. Pretreatment tomograms reveal an asymmetric condyle position; the non-crossbite side is down and forward in the fossa, whereas the crossbite side is centered in the fossa [11]. In subsequent craniofacial development, a functional unilateral posterior crossbite leads to increased growth on the non-crossbite side and to impairment in the crossbite side [14] which might result in facial asymmetry [15, 16]. Several methods have been applied to treat the FPXB, these methods mainly depend on expansion of the maxillary arch such as: W arch, quad helix, Haas, hyrax, or removable appliances. However, the removal of occlusal interferences seems to have a significant role in elimination of the functional shift [17].

Regarding the available literature, there were three systematic reviews about the topic of this review. Tsanidis et al. discussed other outcomes to investigate whether the oral functional asymmetry disappeared after early treatment of FPXB or not. Their review focused on muscle thickness, bite force, and chewing cycle. However, it did not provide any results related to the success and relapse rate of treatment, the expansion amount of different appliances, and the mandible deviation. Thus, this review did not evaluate several important clinical aspects of FPXB treatment [18]. Agostino et al. reviewed 15 RCTs about treatment of posterior unilateral or bilateral crossbite at different ages (8 to 16 years old). Although this review assessed different methods of posterior crossbite correction, some trials included a mixture of patients with and without functional mandibular shifts, which could have affected the estimated treatment outcomes. Additionally, the age groups in the retrieved papers were not consistent among studies, i.e., some trials included adolescent patients instead of being confined to preadolescent age groups [19]. Caroccia et al. focused in their systematic review on unilateral posterior crossbite treatment in primary and early mixed dentition by different types of appliances. Although some of the included trials had the same design and applied similar appliances for expansion, there was no quantitative synthesis of the collected data (i.e., no results of a meta-analysis). Besides, some of the included trials did not clearly state if their patients had a functional shift on closure [20].

Therefore, the aim of this systematic review was to critically appraise the available evidence of the effectiveness of early intervention of functional unilateral posterior crossbites between the ages of 6 and 12 years.

## Materials and methods

Primarily, a PubMed pilot search was carried out. Registration with PROSPERO was performed during the first stages of this review (https://www.crd.york.ac.uk/prospero/display_record.php?ID=CRD42021252830).

This systematic review was written according to the Cochrane Handbook for Systematic Reviews of Interventions 2nd edition [21] and the Preferred Reporting Items for Systematic Reviews and Meta-Analyses (PRISMA) guidelines [22].

### Eligibility criteria

The including and excluding criteria were employed with reference to PICOS framework. Regarding the targeted 'population', patients in mixed dentition period with functional unilateral posterior crossbite associated with mandibular midline deviation were chosen. With regard to the 'intervention', any interceptive treatment such as slow or rapid maxillary expansion by fixed or removable appliances was accepted for inclusion. The 'comparison' group was based on patients receiving any expansion appliance (not used in the interventional group) or a non-treated or a normal control group. The 'outcomes' of interest were: the width of the maxillary and mandibular dental arches, success rate and relapse, treatment duration, correction of mandibular midline. All the included studies were randomized controlled trials (RCTs) or clinical controlled trials (CCTs) written in the English language only.

### Search strategy

An electronic literature search was carried out by two independent reviewers (DHA, SGM) using the following databases: PubMed, *Scopus*®, EMBASE®, and Google Scholar search for the studies published from 1 January 1990 to 31 October 2021. The keywords used and the details of the search strategy are provided in Table 1. References list of selected articles were hand-searched to find any other potentially relevant articles that might not appear in the electronic search.

**Table 1:** Electronic search strategy

**PubMed** Publication date: From 1 January 1990 to 31 October 2021 Search builder: All fields	#**1** (mixed dentition ''OR'' children ''OR'' functional posterior cross bite ''OR'' posterior cross bite ''OR'' unilateral cross bite ''OR'' lateral cross bite ''OR'' mandibular shift ''OR'' lateral shift**) #2 (**expansion ''OR'' maxillary expansion ''OR'' maxilla expansion ''OR'' interceptive treatment ''OR'' interceptive therapy ''OR'' interceptive orthodontics) **#3 (intermolar width ''OR'' intercanine width ''OR'' arches width ''OR'' transvers width ''OR'' midline correction)** **#4** #1 AND #2 AND #3
**Scopus** Publication date: From 1 January 1990 to 31 October 2021	**#1** TITLE-ABS-KEY (mixed dentition ''OR'' children ''OR'' functional posterior cross bite ''OR'' posterior cross bite ''OR'' unilateral cross bite ''OR'' lateral cross bite ''OR'' mandibular shift ''OR'' lateral shift) **#2** TITLE-ABS-KEY **(**expansion ''OR'' maxillary expansion ''OR'' maxilla expansion ''OR'' interceptive treatment ''OR'' interceptive therapy ''OR'' interceptive orthodontics) **#3** TITLE-ABS-KEY (intermolar width ''OR'' intercanine width ''OR'' arches width ''OR'' transvers width ''OR'' midline correction) **#4** #1 AND #2 AND #3
**EMBASE** Publication date: From 1 January 1990 to 31 October 2021	#**1** (mixed dentition ''OR'' children ''OR'' functional posterior cross bite ''OR'' posterior cross bite ''OR'' unilateral cross bite ''OR'' lateral cross bite ''OR'' mandibular shift ''OR'' lateral shift**) #2 (**expansion ''OR'' maxillary expansion ''OR'' maxilla expansion ''OR'' interceptive treatment ''OR'' interceptive therapy ''OR'' interceptive orthodontics) #3 (intermolar width ''OR'' intercanine width ''OR'' arches width ''OR'' transvers width ''OR'' midline correction) **#4** #1 AND #2 AND #3
**Google Scholar** Publication date: From 1 January 1990 to 31 October 2021	**#1** (mixed dentition ''OR'' children ''OR'' functional posterior cross bite ''OR'' posterior cross bite ''OR'' unilateral cross bite ''OR'' lateral cross bite ''OR'' mandibular shift ''OR'' lateral shift) AND **(**expansion ''OR'' maxillary expansion ''OR'' maxilla expansion ''OR'' interceptive treatment ''OR'' interceptive therapy ''OR'' interceptive orthodontics) AND (intermolar width ''OR'' intercanine width ''OR'' arches width ''OR'' transvers width ''OR'' midline correction)

### Study selection and data extraction

Two reviewers (DHA, SGM) carried out the studies selection independently in accordance with eligibility criteria, and in case of disagreement, a third author (MYH) was asked to resolve this. Initially, all potential articles were screened according to the title and abstract. Then the full text of all selected articles was checked, and the final selection was made based on the pre-defined selection criteria. The following data were extracted from each of the included articles: general information (Authors name, publication date), study design, number of groups, size and the mean age of samples, intervention (type of appliance), treatment and retention time, and outcomes.

### Assessment of risk of bias in individual studies

The quality of the included studies was assessed by two reviewers (DHA and SGM), When lack of consistency was observed, a third author (MYH) was consulted to arrive at a resolution. The reviewers used Cochrane's risk of bias tool (ROB1) for RCTs as a judgment (high, low, or unclear) for individual elements from five domains (selection, performance, attrition, reporting, and other) [23]. The overall risk of bias of the individual studies was evaluated. Low risk of bias was considered when all fields were assessed as at low risk of bias. Unclear risk of bias was considered when one or more fields were assessed as at unclear risk of bias. High risk of bias was considered when one or more fields were evaluated as at high risk of bias. The MINORS tool [24] was used to assess non-randomized studies (CCTs). The MINORS tool was analyzed through 12 items. Four of them are additional in case of comparative studies. The items are scored 0 (not reported), 1 (reported but inadequate,) or 2 (reported and adequate). The global ideal score is 16 for non-comparative studies and 24 for comparative studies.

### Data synthesis, assessment of risk of bias across studies, and assessment of strength of evidence

Meta-analysis was carried out using Review Manager, Version 5.3. Copenhagen: The Nordic Cochrane Centre, the Cochrane Collaboration. Heterogeneity was first evaluated visually and then mathematically. Two reviewers (DHA and MYH) checked the graphical display of the estimated treatment effects with 95% confidence intervals. Then, The *P* value was calculated to discover any significant heterogeneity when *P* < 0.05. *I*^2^ index was used to describe the percentage of heterogeneity across the studies, and its values were explained as follows: low heterogeneity: 0–40%, moderate to high heterogeneity: 30–60%, significant heterogeneity: 50–90%, and very significant heterogeneity: 75–100% [25]. Data were pooled to meta-analysis when trials had comparable interventions, subjects, and outcomes. Mean differences (MD) with their associated 95% confidence intervals (CI) were chosen to express results as effect measure in case of continuous outcomes and the risk ratio (RR) was chosen in case of dichotomous outcomes. The treatment effect was weighted (weighted mean difference (WMD) using calculations based on a random-effects model; this model was considered appropriate because of the noticed differences in settings and populations. The inverse-variance method was chosen in cases of continuous outcome measures and Mantel–Haenszel statistical method was applied for dichotomous outcome measures. All of the mean and standard deviation of the differences were extracted directly from the included studies. When there was a need to combine subgroups and obtain the mean and standard deviation of the combined data in any of the included RCTs, Review Manager's calculator has been used to attain the combined means and SDs. The forest plots were used to present a graphical assessment of the analysis results. Sensitivity analysis was conducted by tracing sensitivity plots to investigate the influence of the CCTs on the results and omitting them when appropriate. The publication bias was planned to be assessed throughout the funnel plots when ten or more trials were collected for quantitative synthesis. The certainty of the evidence was evaluated according to grading of recommendations assessment, development, and evaluation (GRADE) approach as follows: high certainty, moderate certainty, low certainty, and very low certainty [26]. The GRADEpro GDT was used to evaluate the evidence and to get the 'summary of findings table' [27].

## Results

### The flow of the search strategy

**Fig. 1:**
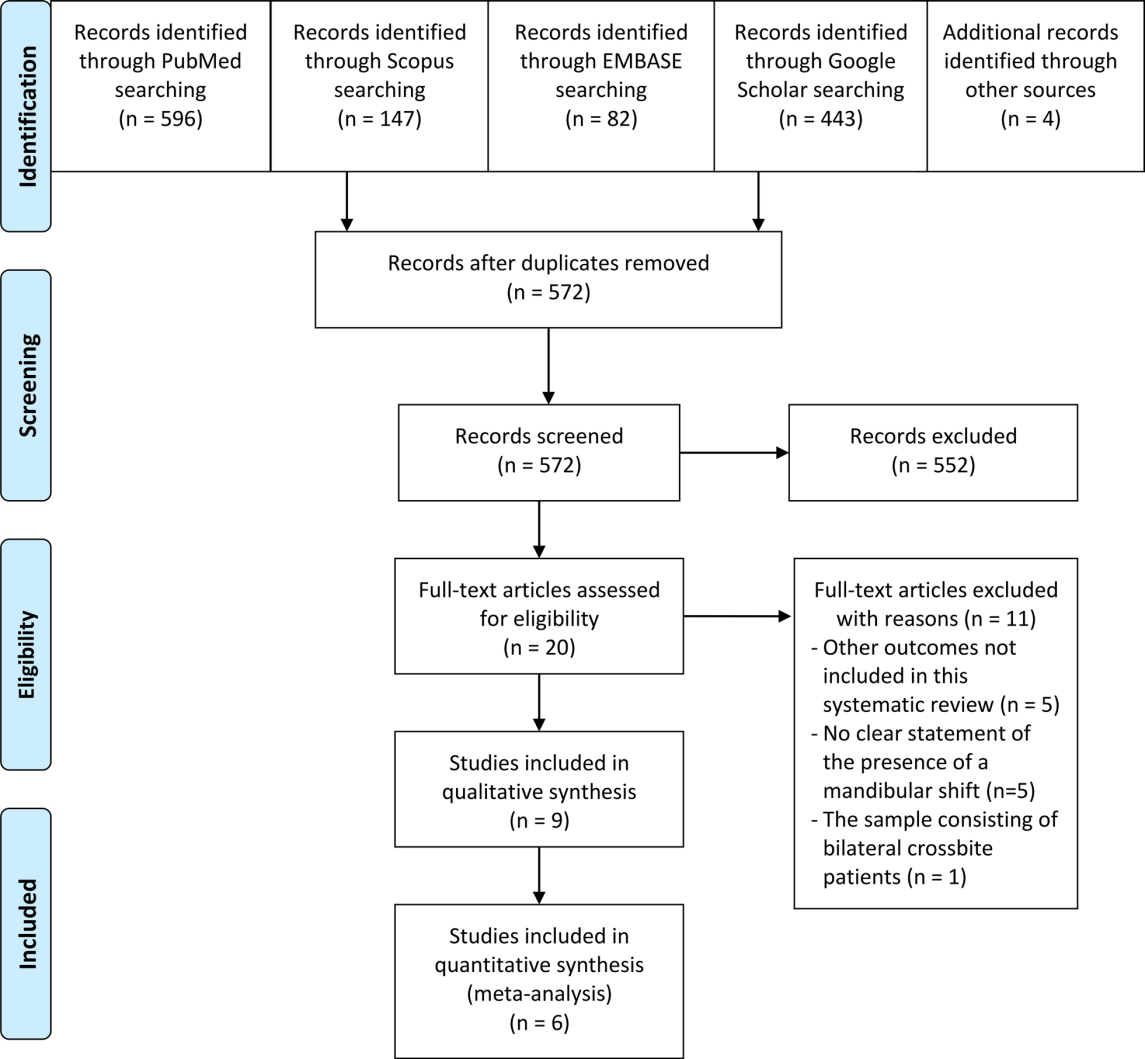
PRISMA flow diagram

The electronic literature search in PubMed, Scopus*®*, EMBASE®, and Google Scholar identified a total of 1272 articles. In addition, four articles were obtained by hand-search in references list of selected articles. Duplicated articles were taken off and a total of 572 were checked. Eleven articles were excluded after the full-text assessment [28–38]. Additional file 1: Table S 1 shows the excluded articles with the principal reason for exclusion. The total included studies were nine; six of them were included in the quantitative synthesis (meta-analysis). Figure 1 shows PRISMA flow diagram.

### Characteristics of the included studies

The included studies in this systematic review were nine studies (6 RCTs, 3 CCTs). All of them included patients in the mixed dentition period aged between 6.9 and 10.2 years. Eight studies measured the intercanine and intermolar width [6, 39–45]. Regarding the correction of the mandibular midline and the rate of success and/or relapse, these outcomes were assessed in 5 studies [40, 42–44, 46], and 7 studies [6, 40–43, 45, 46], respectively. All the studies followed the parallel-group design. Two-arm trials comprised three of them and the three-group design was found in other three studies [6, 41, 43]. Four-group or more designs were encountered in another three studies [42, 45, 46].

Eight studies (89% of the all included studies) had control groups of untreated subjects [6, 42, 44, 45] or normal occlusion subjects [40, 41, 43, 45]. Only one study had treated control group [39]. Quad-helix-based expansion therapy was undertaken in seven studies, and jackscrew-based removable appliances were used in eight studies. There was only one study, which evaluated rapid maxillary expansion [44]. However, there was no study comparing slow and rapid expansion in the treatment of FPXB in the mixed dentition period. Boysen et al. and Petern et al. 2008 compared two appliances of slow expansion (quad-helix and expansion plates) [39, 43], whereas Lippold et al. compared rapid expansion by bonded Hyrax appliance with a group of untreated patients [44]. The three-arm design studies compared the quad-helix and the removable plate against a control group of normal occlusion or untreated patients [6, 41, 43]. One of the four-arm design studies compared all of the following: quad-helix, removable expansion plates, composite onlay, and untreated control group [42]. The other four-arm study compared QH versus EP from two different panels of health care providers (general dentists versus orthodontists) [46]. The comparison was undertaken between six groups in only one study, which included untreated group and normal occlusion control group [45].

The quad-helix design was almost similar in all trials. The expansion plate had a similar design which did not include an acrylic covering of the posterior occlusal surfaces in seven studies. However, there was only one study, which applied a posterior acrylic bite plane with the expansion plates [39]. The activation protocol of appliances differed from one study to another. The quad-helix was activated only for one time before bands cementation in one study, and the patients did not need any extra expansion [39]. However, in six studies the activation was performed once every four or six weeks till the crossbite corrected with a transverse activation ranging from 3 to 10 mm for the first time [6, 41–43, 45, 46]. The expansion protocol for removable expansion plates was not identical among the included trials. Although five studies (62% of all studies used EP) applied an expansion protocol with one turn per week (i.e., ≈ 0.2 mm; one-quarter of a full turn) [6, 42, 43, 45, 46], two studies used a quicker expansion plan with a 0.5 mm expansion per week [39, 41]. The characteristics of the nine included studies can be found in Table 2. All the outcomes' measurements are shown in Table 3.

**Table 2:** Characteristics of the included trials

References	Participants	Treatment/retention/observation time	Expansion obtained on the maxillary arch	Success rate and relapse	Correction of midline /mandibular width/other outcomes
Boysen et al. [39] CCT	34 children UFPXB: G1 (8.3 Y): 17 QH G2 (8.6 Y): 17 EP	G1 (QH): treatment duration 101.2 D G2 (EP): treatment duration 115.4 D Retention: 3 M Observation: 2 Y	IC: QH 5.1 mm EP 3.5 mm** IM: QH 5.6 mm EP 4.6 mm*		Mandibular IM: decreased in QH (− 0.02 mm) Increased in EP (0.02 mm)* Mandibular IC: increased in QH (0.01 mm) Decreased in EP (− 0.16 mm)*
Brin et al. [40] CCT	34 children: TG (9.5 Y): 24 (UFPXB) EP CG (9.8 Y): 10 normal	TG (EP): duration 10 M Retention 6 M	IM increased in EP (3 mm) and became similar to the CG	Success rate: EP 50% No relapse after 6 M	Midline deviation correction:95% The mandibular arch width in TG: decreased (25% of the cases); increased (50%); remained the same (25%)
Bjerklin et al. [41] CCT	38( FPXB): G1 (9.3 Y): 19 QH G2 (9.2 Y):19 EP CG (8.8 Y): 19 normal	G1 (QH): 7.7 M G2 (EP): 12.5 M Retention 3–5 M Observation 5.5 Y	IC: (QH): 1.6 mm (EP): 2.3 mm* IM: (QH): 3.6 mm (EP): 2.9 mm*	Success rate in both groups was 100% Relapse (after 5.5 Y): (QH) 3/19 (EP) 1/19	No differences in the mandibular arch
Petren et al. [42] RCT	60( FPXB): A (9.1 Y): 15 QH B (8.7 Y): 15 EP C (8.3 Y): 15 composite onlay D (8.8 Y): 15 CG	Group A: 4.8 M Group B: 9.6 M Group C: 1 Y Retention: Groups A and B:6 M Observation: 1 Y	IC: A: 1.4 mm B: 2.4 mm** C: 0.5 mm D: 0.2 mm* IM: A: 4.6 mm B: 3.5 mm** C: 0.5 mm D: 0.4 mm*	Success rate A: 15/15 (100%) B: 10/15 (66.6%)** C: 2/15 (13.3%) D: 0/15 (0%)*	Midline deviation correction: A:14/15 B:12/15 C:6/15 D:3/15 Mandibular arch changes: IC (*) IM ( increased in B & D groups)
Godoy et al. [6] RCT	99 (FPXB): G1 (8 Y): 33 QH G2 (7.8 Y): 33 EP G3 (8.09 Y): CG	QH: 4.24 M EP: 6.12 M Retention: 6 M Observation: 12 M (after cross bite correction)	IC: (QH): 4.5 mm (EP): 1.8 mm* IM: (QH): 5.7 mm (EP): 4.46 mm*	Success rate (QH):100% (EP):90.9% (CG): 0% Relapse: (QH): 9.1% (EP): 9.1%	Mandibular arch changes: IM: QH > EP & CG Side effects occurred in: (QH):39.4% (EP): 27.3%
Petren et al. [43] RCT	40 (PXB) with midline deviation: A(9 Y): 20 QH B (8.5 Y): 20 EP (withdrawal of 5 patients later) CG (8.8 Y): 20 normal	Treatment duration: 1Y Retention: 6 M Observation: 3 Y	IC: A: 2.7 mm B: 2.6 mm*IM: A: 4.1 mm B: 3.8 mm*	Relapse after 3 Y: (QH): ½0 (EP): 0/15*	Midline deviation: correction occurred in > 50% in both groups with no significant differences Mandibular arch changes: IC:* IM: B > A
Lippold et al. [44] RCT	77 (FPXB): A (8.3 Y): 37 bonded Hyrax followed by a U-bow activator ( withdrawal of 6 patients later) B (8.2 Y): 40 CG ( withdrawal of 5 patients later)	Bonded Hyrax: 3.2 W Retention: 12.6 W U-bow activator: 36.8 W	IC: A: 2.6 mm B: 1 mm** IM:A: 5.1 mm B: 0.8 mm**		midline correction: A > B Mandibular arch changes:*
Sollenius et al. [45] RCT	135 UFPXB: 1.QHS (9.3Y): 28 QH in SOC 2. QHG (9.5Y): 27 QH in GDC 3.EPS(8.7Y): 27 EP in SOC 4. EPG (9.2Y): 28 EP in GDC 5.Untreated group (8.5Y): 25 CG (9.3Y): 25 normal	Treatment duration: QHS: 7.5 M QHG: 8.2 M EPS: 11.4 M EPG: 12 M Including 3 M for retention	IC: QHS > QHG & EPS & EPG** IM: QHS > QHG & EPS & EPG**	Success rate: QHS: 100% QHG: 85.1% EPS: 66.6% EPG: 64.2% Treatment by QHS was significantly more successful compared to other groups	
Sollenius et al. [46] RCT	110 (FPXB): 1. QHS (9.3Y): 28 QH in SOC 2. QHG (9.6Y): 27 QH in GDC 3. EPS (8.8Y): 27 EP in SOC 4. EPG (9.0Y): 28 EP in GDC	Number of appoint QHS: 7.1 QHG: 8.4 EPS: 8.2 EPG: 9.2 Including 3 M of retention		Success rate: QHS: 100% QHG: 85.18% EPS: 66.6% EPG: 64.28%	midline correction: QHS: 85.71% QHG: 50% EPS: 59.25% EPG: 50%

UFPXB: unilateral functional posterior cross bite, QH: quad helix, EP: expansion plate, CG: control group, TG: treatment group, RME: rapid maxillary expansion, IC: intercanine width, IM: intermolar width, SOC: specialist orthodontic clinics, GDC: general dentistry clinics, CO: centric occlusion, MO: maximum occlusion, XB: crossbite, N-XB: non-crossbite

**Statistically significant difference, *Non-statistically significant difference

**Table 3:** The quad-helix versus the expansion plates (pre and post-expansion differences in the evaluated outcomes) and the RME versus an untreated CG (pre and post-treatment measurements of the evaluated outcomes)

References	Comparison between QH and EP Outcomes changes (T2-T1)							
	Maxillary intermolar width [mm]	Maxillary intercanine width [mm]	Mandibular intermolar width [mm]	Mandibular intercanine width [mm]	Success rate [ratio]	Relapse rate at different time points [ratio]	Treatment duration [months]	Correction of the mandible midline [ratio]
Boysen et al. [39]	QH: 5.61 ± 1.78 EP: 4.65 ± 1.52	QH: 5.17 ± 1.86 EP: 3.50 ± 1.25	QH: − 0.20 ± 2.32 EP: 0.02 ± 0.36	QH: 0.01 ± 0.74 EP: − 0.16 ± 0.64	Not-studied	Not-studied	Not-studied	Not-studied
Bjerklin et al. [41]	QH: 1.2 ± 0.67 EP: 1.5 ± 1.04	QH: 1.6 ± 1.04 EP: 2.3 ± 1.22	QH: 0.0 ± 0.21 EP: 0.0 ± 0.57	QH: 0.1 ± 0.23 EP: − 0.1 ± 0.91	QH: 19/19 EP: 19/19	Not-studied	QH: 7.7 ± 2.79 EP: 12.5 ± 4.22	Not-studied
Petren et al. [42]	QH: 4.4 ± 1.19 EP: 3 ± 1.57	QH: 2 ± 1.18 EP: 2.7 ± 1.2	QH: − 0.1 ± 0.62 EP: 0.5 ± 0.67	QH: 0.1 ± 0.26 EP: 0.2 ± 0.28	QH: 15/15 EP: 10/15	Not-studied	QH: 4.8 ± 3.52 EP: 9.6 ± 3.04	QH: 14/15 EP: 12/15
Petren et al. [43]	QH: 3.7 ± 1.58 EP: 3.2 ± 1.24	QH: 2.7 ± 1.57 EP: 2.6 ± 1.58	QH: − 0.4 ± 0.82 EP: 0.4 ± 0.67	QH: − 0.5 ± 1.21 EP: 0.5 ± 1.42	Not-studied	Relapse after 3 Y QH: ½0 EP: 0/15	Not-studied	Not-studied
Godoy et al. [6]	QH: 5.7 ± 2.31 EP: 4.46 ± 2.22	QH: 3.48 ± 2.24 EP: 1.8 ± 2.96	QH: 0.46 ± 1.20 EP: − 0.12 ± 1.36	QH: − 0.21 ± 0.92 EP: 0.28 ± 1.51	QH: 33/33 EP: 30/33	Relapse after 1 Y QH: 3/33 EP: 3/33	QH: 4.24 ± 2.05 EP: 6.12 ± 3.25	Not-studied
Sollenius et al. [45]	QH: 4.11 ± 1.86 ^A^ EP: 2.49 ± 1.67 ^A^	QH: 3.36 ± 1.87 ^A^ EP: 2.53 ± 2.81 ^A^	Not-studied	Not-studied	QH: 51/55 ^A^ EP: 36/55 ^A^	Not-studied	QH: 4.84 ± 1.88 ^A^ EP: 8.7 ± 3.49 ^A^	Not-studied
Sollenius et al. [46]	Not-studied	Not-studied	Not-studied	Not-studied	Not-studied	Not-studied	Not-studied	QH:34/48 ^A^ EP: 26/44 ^A^

References	Comparison between RME and untreated CG							
	Measurement time	Maxillary intermolar width [mm]	Maxillary intercanine width [mm]	Mandibular intermolar width [mm]	Mandibular intercanine width [mm]	Correction of the mandible midline [mm]		
Lippold et al. [44]	T1	RME (*n* = 31): 42.2 ± 2.6 CG (–35): 42.6 ± 3.1	RME (*n* = 31): 29 ± 2.6 CG (–35): 27.9 ± 2.2	RME (*n* = 31): 48.1 ± 2 CG (*n* = 35): 47.2 ± 02.5	RME (*n* = 31): 25.8 ± 1.9 CG (*n* = 35): 25.2 ± 1.7	RME (*n* = 31): 2.1 ± 1.3 CG (*n* = 35): 1.9 ± 1.2		
	T2	RME (*n* = 31): 47.3 ± 2.5 CG (–35): 43.4 ± 2.3	RME (*n* = 31): 32.6 ± 2.7 ^A^ CG (–35): 28.9 ± 2.2	RME (*n* = 31): 48.6 ± 1.7 CG (*n* = 35): 47.7 ± 2.6	RME (*n* = 31): 25.9 ± 1.7 CG (*n* = 35): 25.4 ± 1.6	RME (*n* = 31): 0.5 ± 0.5 CG (*n* = 35): 2.1 ± 1.3		

QH: quad-helix, EP: expansion plate, T1: before expansion, T2: after expansion, A: the mean and SD for the combined subgroups were calculated mathematically depending on certain formulas. CO: centric occlusion, MO: maximum occlusion, XB: crossbite, N-XB: non-crossbite, RME: rapid maxillary expansion, CG: control group

### Risk of bias in individual studies and across studies

**Fig. 2:**
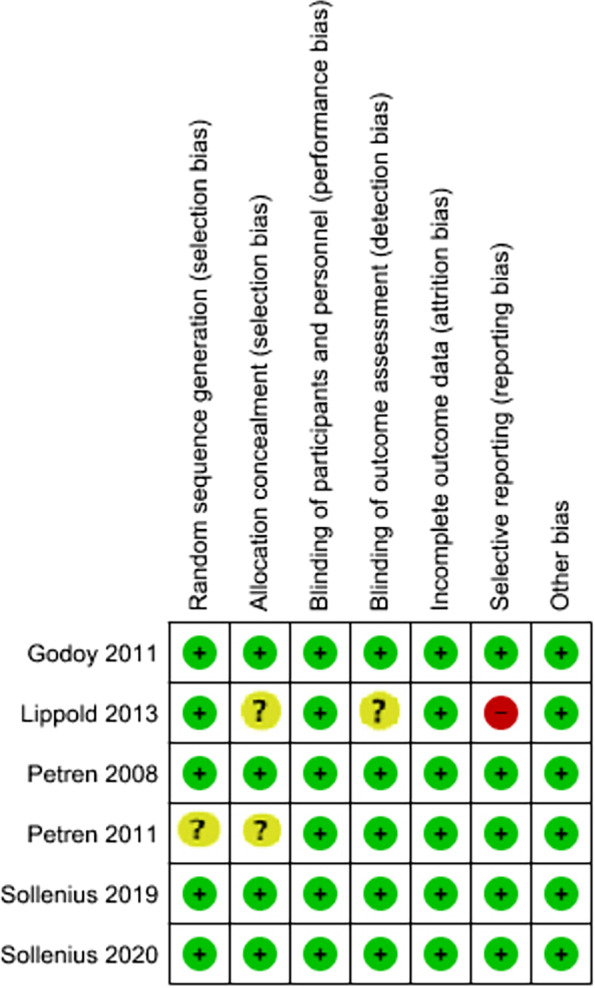
Risk of bias summary: review authors' judgements about each risk of bias item for each included study

Figure 2 and Table 4 show the risk of bias of the individual studies. There were four RCTs assessed as 'low risk of bias' [6, 42, 45, 46]. One RCT was judged to be at 'high risk of bias', and the other one was evaluated as 'unclear risk of bias'[43]. The quality assessment is shown in Additional file 2: Table S 2. The random sequence generation and allocation concealment were unclear in 25% and 50% of the studies, respectively. There was a detection bias in 25% of the included RCTs. The selective reporting field was evaluated as 'high risk' in 25% of the RCTs. According to the MINORS tool that was applied for three studies [39–41], the most problematic fields were the assessment of the study endpoint and prospective calculation of the study sample size. The least risk of bias was found in Bjerklin et al. study [41]. The other two studies scores were 17/24 [39, 40], which showed that the included CCTs were of fair quality. The publication bias was not assessed because we did not collect ten studies. Therefore, the funnel plots were not used in this meta-analysis.

**Table 4:** Risk of bias for non-randomized studies according to the MINORS tool (20)

References	1. A clearly stated aim	2. Inclusion of consecutive patients	3. Prospective collection of data	4. Endpoints appropriate to the aim of the study	5. Unbiased assessment of the study endpoint	6. Follow-up period appropriate to the aim of the study	7. Loss to follow up less than 5%	8. Prospective calculation of the study size	9. An adequate control group	10. Contemporary groups	11. Baseline equivalence of groups	12. Adequate statistical analyses	Total
Boysen et al. [39]	2	1	2	2	0	2	0	0	2	2	2	2	17/24
Brin et al. [40]	2	2	2	2	0	2	1	0	1	1	2	2	17/24
Bjerklin et al. [41]	2	2	1	2	0	2	2	0	2	2	2	2	19/24

### Effects of interventions

According to the available trials in this systematic review, two main themes of comparisons could be made. The first set of comparisons was between the quad-helix and the expansion plates in terms of primary and secondary outcomes, whereas the second set of comparisons was restricted to the differences between the rapid maxillary expansion and the untreated control groups.

## First: The quad-helix versus the removable expansion plate

### Widths of the maxillary and mandibular dental arches

**Fig. 3:**
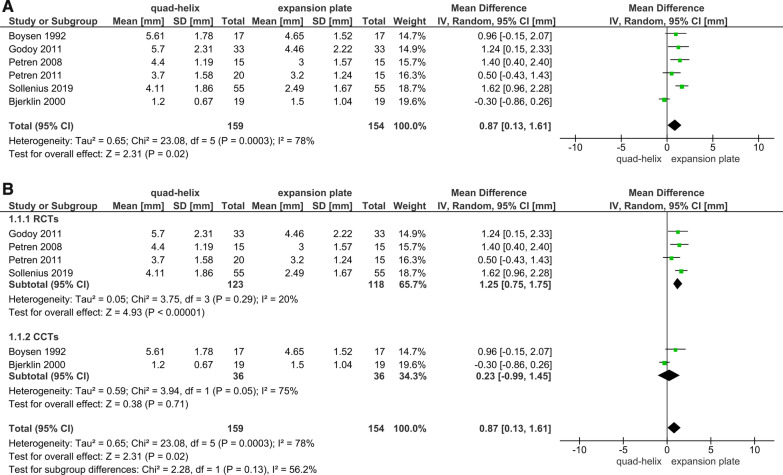
Forest plot of comparison: comparison between quad-helix and expansion plate, outcome: maxillary intermolar width [mm] without sensitivity analysis (**A**) and with sensitivity analysis (**B**)

**Fig. 4:**
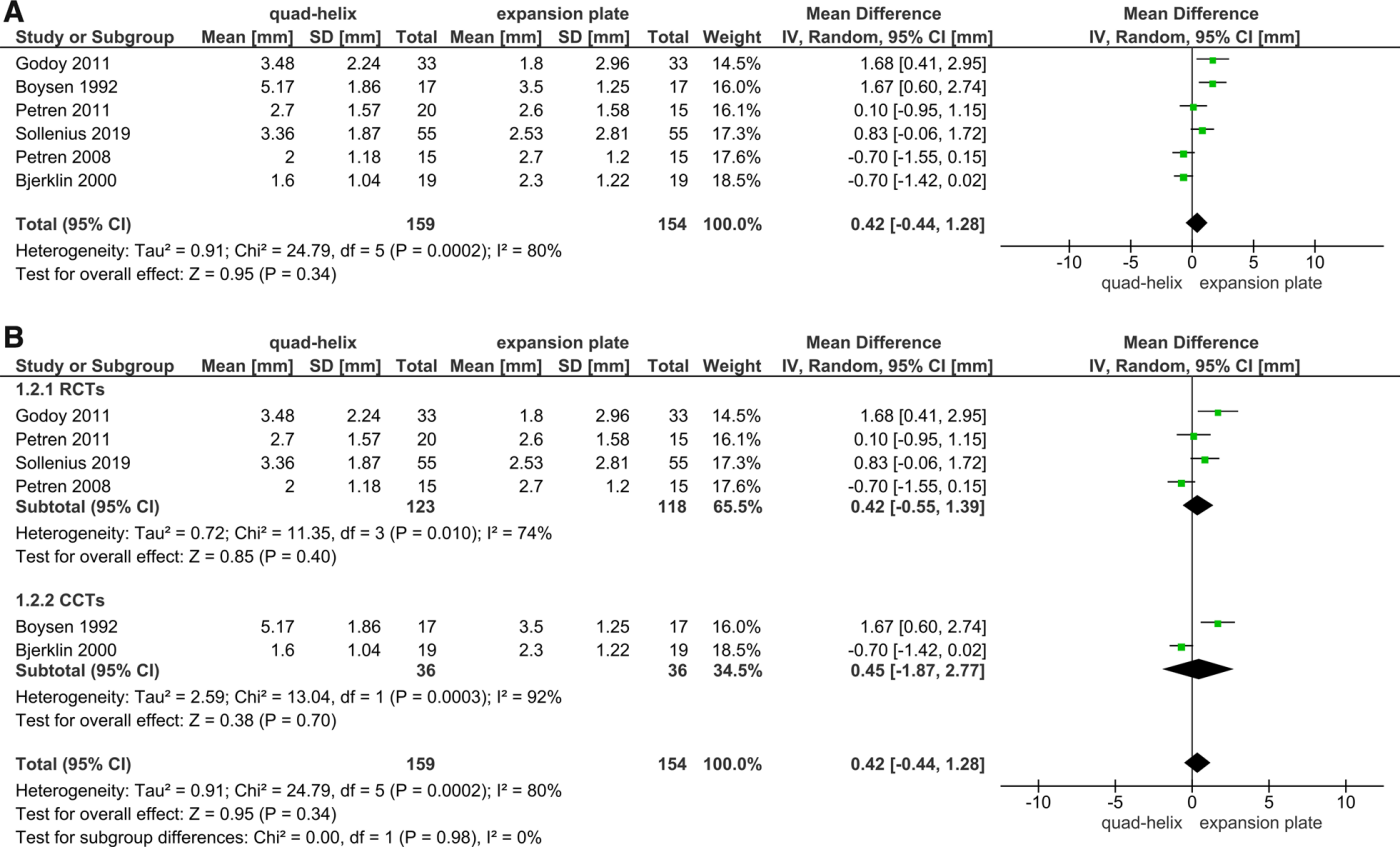
Forest plot of comparison: comparison between quad-helix and expansion plate, outcome: maxillary intercanine width [mm] without sensitivity analysis (**A**) and with sensitivity analysis (**B**)

**Fig. 5:**
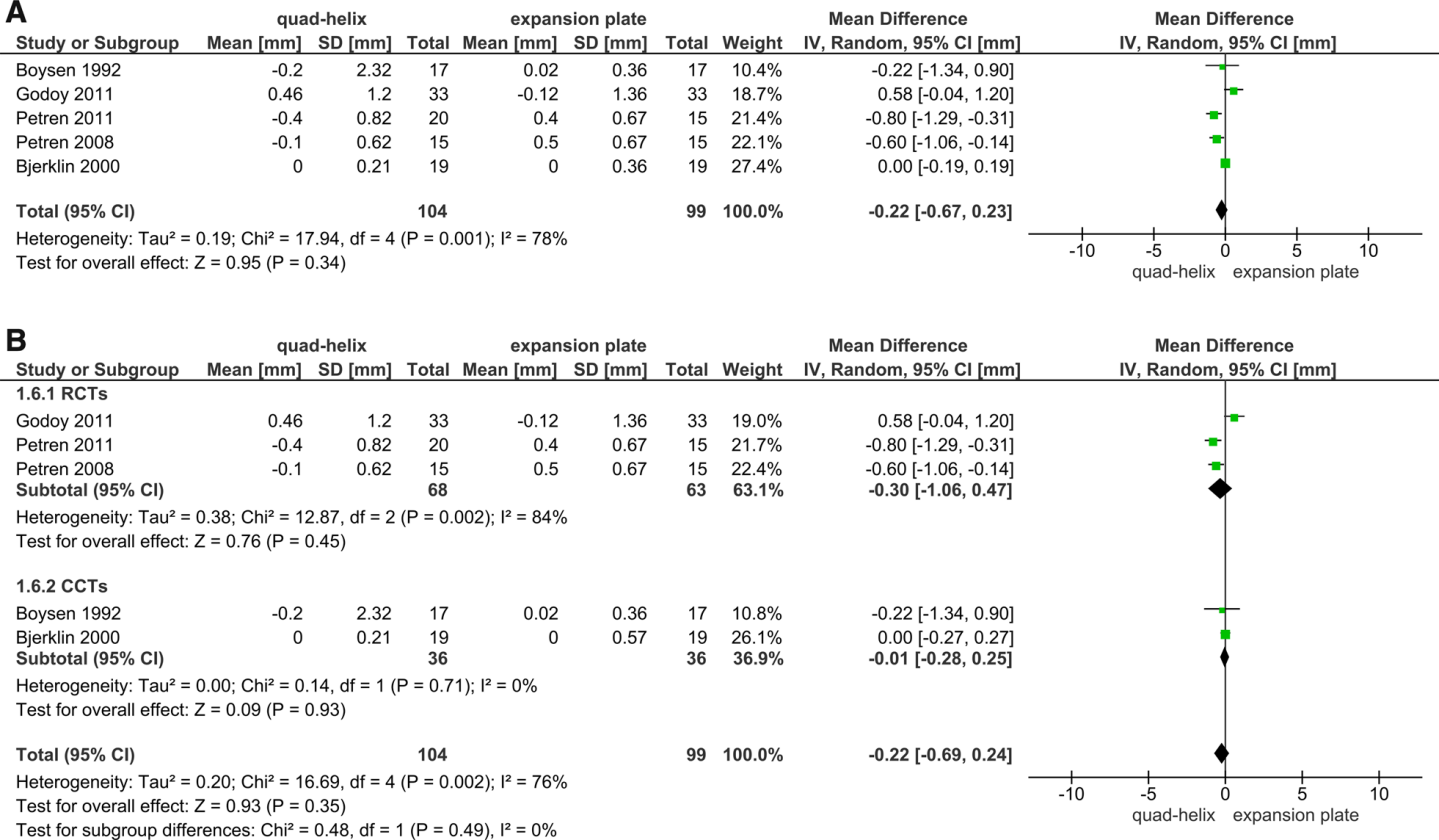
Forest plot of comparison: comparison between quad-helix and expansion plate, outcome: mandibular intermolar width [mm] without sensitivity analysis (**A**) and with sensitivity analysis (**B**)

**Fig. 6:**
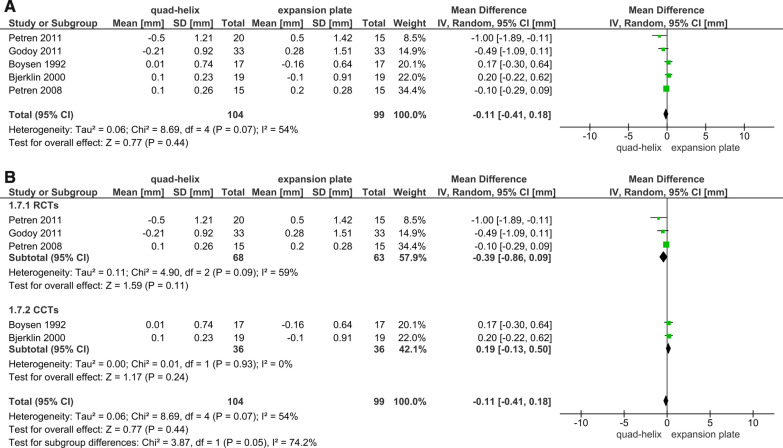
Forest plot of comparison: comparison between quad-helix and expansion plate, outcome: mandibular intercanine width [mm] without sensitivity analysis (**A**) and with sensitivity analysis (**B**)

Six articles (4 RCTs and 2 CCTs) studied the dental arch dimensions and the differences caused by expansion [6, 39, 41–43]. All the measurements were extracted from the trials immediately after the desired expansion has been achieved. The sensitivity analysis was conducted in all outcomes related to arches^'^ dimensions. It was shown that the included CCTs in the primary analyses had affected the overall results due to a significant change in *P* value when sensitivity analyses were applied (Figs. 3A, 4A, 5A, 6A). The heterogeneity coefficient (*I*^2^) also differed after sensitivity analyses were achieved, which may affect the evaluation of inconsistency during the assessment process of the level of evidence. Therefore, it was decided not to include CCTs with RCTs for more reliable results.

A greater increase in the maxillary intermolar width was found in quad-helix group compared to the removable expansion plate group, and the difference was statistically significant (WMD = 1.25; 95% CI 0.75–1.75; *P* < 0.001; Fig. 3B). Heterogeneity was low (Chi^2^ = 3.75, *df* = 3, *P* = 0.29; *I*^2^ = 20%). For the maxillary intercanine width, there was a non-significant difference between two groups (WMD = 0.42; 95% CI − 0.55, 1.39; *P* = 0.40; Fig. 4B). However, for that measure, there was a significant heterogeneity between the included studies (Chi^2^ = 11.35, *df* = 3, *P* = 0.01; *I*^2^ = 74%). Regarding the mandibular arches' dimensions and the changes occurred due to maxillary expansion by either QH or EP; the pooled estimate showed a non-statistically significant difference in the mandibular intermolar and intercanine width between QH and EP (*P* = 0.45; 0.11, respectively). There was also a very significant heterogeneity for the mandibular intermolar width outcome (Chi^2^ = 12.87, *df* = 2 (*P* = 0.002); *I*^2^ = 84%; Fig. 5B); and a moderate heterogeneity for the mandibular intercanine width outcome (Chi^2^ = 4.90, *df* = 2 (*P* = 0.09); *I*^2^ = 59%; Fig. 6B).

### Success and relapse rate

**Fig. 7:**
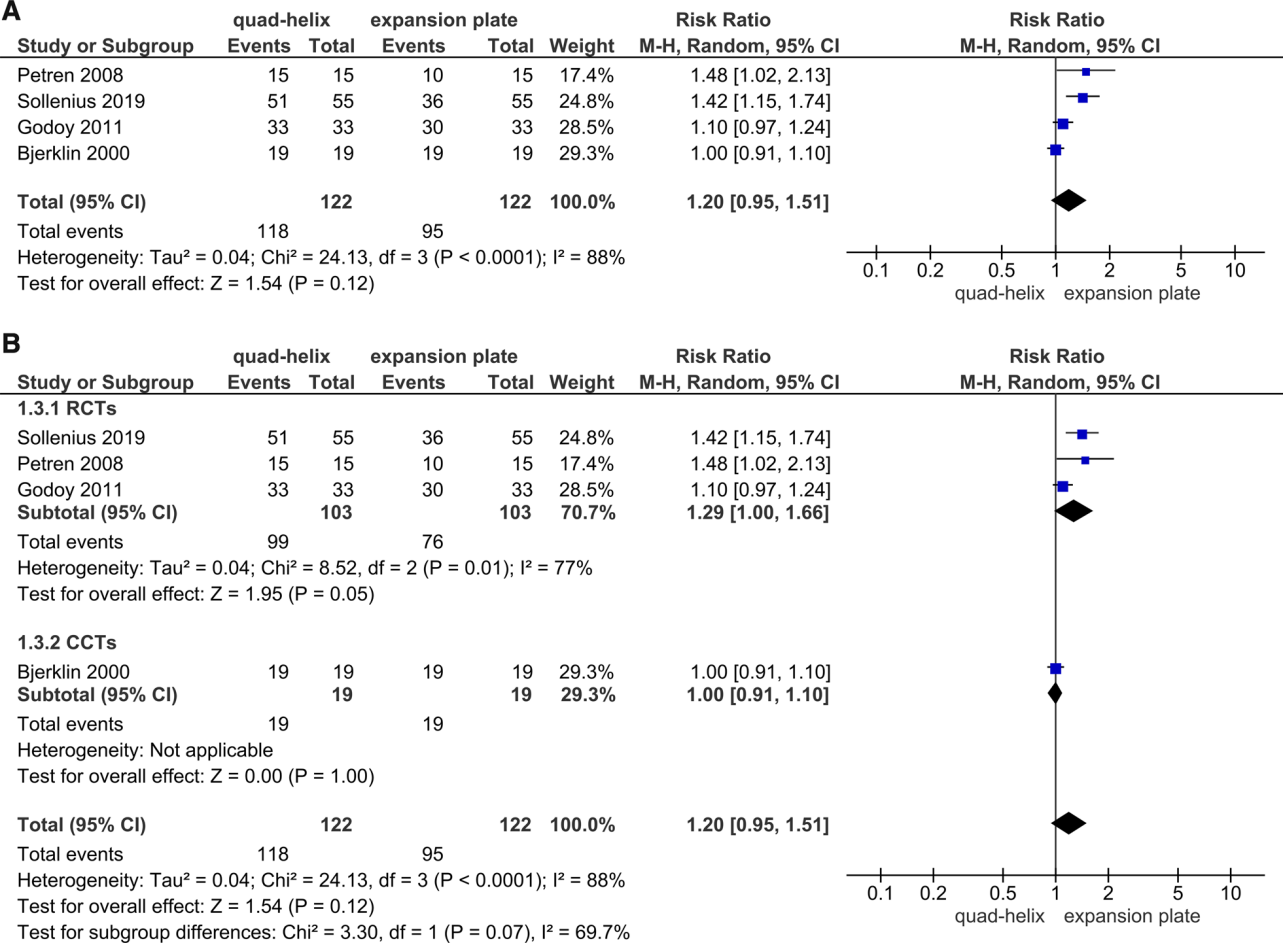
Forest plot of comparison: comparison between quad-helix and expansion plate, outcome: success rate [ratio] without sensitivity analysis (**A**) and with sensitivity analysis (**B**)

Four studies (3 RCTs, 1 CCT) assessed the success rate of treatment [6, 41, 42, 45]. The primary analysis showed a non-significant difference between QH and EP groups (*P* = 0.12, Fig. 7A). However, after the sensitivity analysis was applied, the overall effect revealed a significant difference between the two groups (*P* = 0.05). Therefore the CCT was not included to attain more accurate results. The heterogeneity was significant for that outcome (Chi^2^ = 8.52, *df* = 2, *P* = 0.01; *I*^2^ = 77%; Fig. 7B). Regarding the relapse, there were two RCTs and one CCT assessing this outcome at different time points which prevented the possibility of conducting a meta-analysis. The first RCT by Godoy et al. evaluated relapse after one year of FPXB correction [6]. It showed that about 9% of patients had relapse in both the QH and EP groups with no difference between them (*P* ≈ 1.00). The other RCT by Petren et al. followed up the included patients for 3 years and the relapse rate was 5% in QH group and 0% in EP group with non-significant difference between the two groups (*P* = 0.612) [43]. After 5.5 years of expansion by either QH or EP, the relapse was seen in 15.7% and 5.2% of patients, respectively, in the CCT of Bjerklin et al. [41], however, the difference between both groups was insignificant.

### Treatment duration

**Fig. 8:**
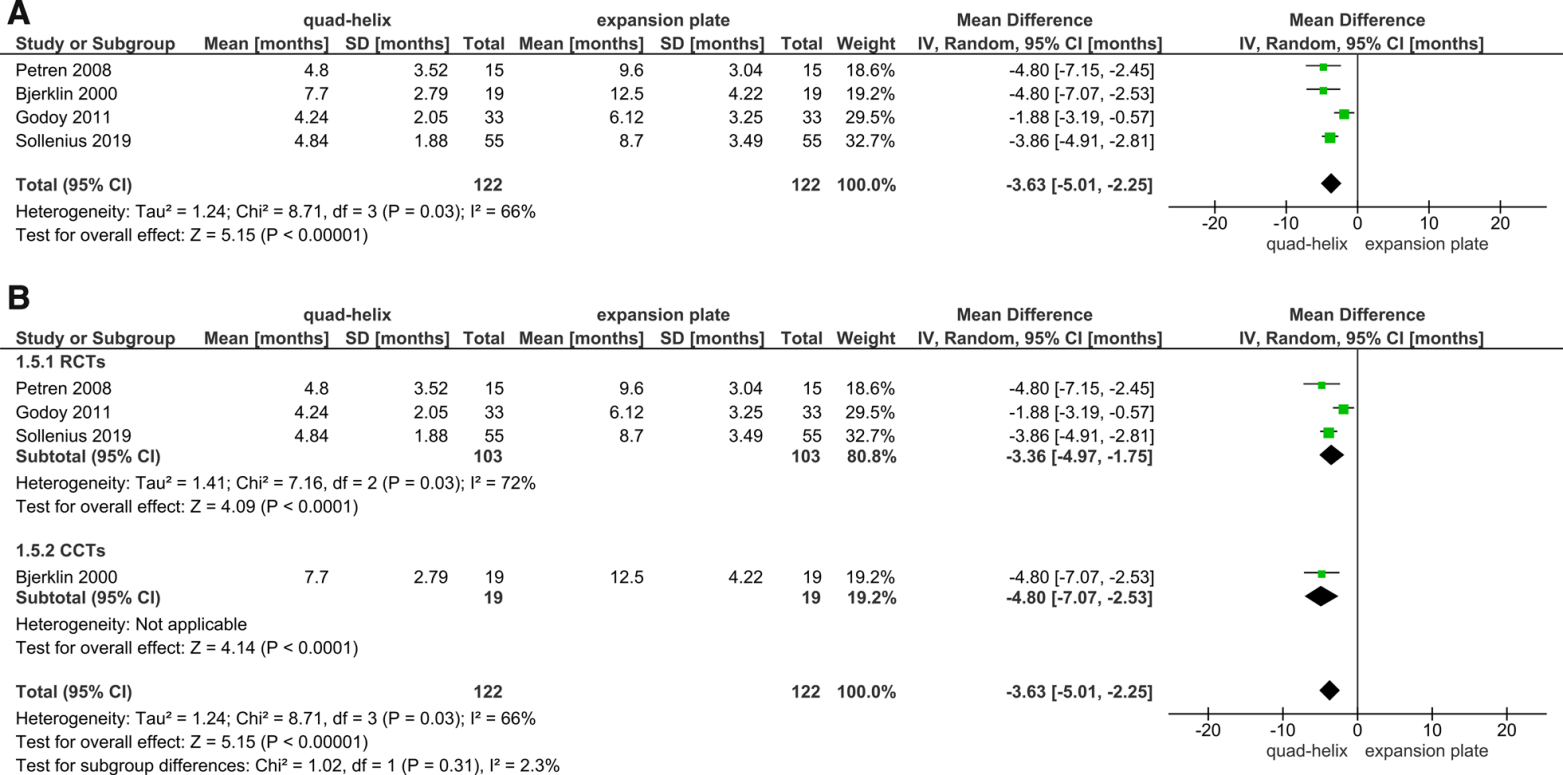
Forest plot of comparison: comparison between quad-helix and expansion plate, outcome: treatment duration [months] without sensitivity analysis (**A**) and with sensitivity analysis (**B**)

The treatment duration by quad-helix ranged from 3.36 to 7.7 months [6, 39, 41, 42], whereas the expansion by removable expansion plate took from 3.8 to 12.5 months [6, 39–42, 45]. There were 3 RCTs and 1 CCT comparing the treatment duration between the QH and the EP. It was decided not to include the CCT in the meta-analysis due to the outcome of running the sensitivity analysis (Fig. 8A). The pooled estimate effect of the three included RCTs showed that quad-helix reduced the treatment duration by an average of 3.36 months (95% CI − 4.97, − 1.75) compared with expansion plate; which was statistically significant (*P* < 0.001; Fig. 8B).

### Correction of the mandibular midline deviation

**Fig. 9:**
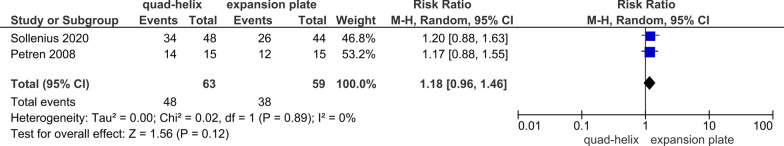
Forest plot of comparison: comparison between quad-helix and expansion plate, outcome: mandibular midline corrections [ratio]

Only two RCTs were deemed appropriate to be included in meta-analysis [42, 46]. The meta-analysis revealed that there was a non-significant difference between the QH and EP patients in correcting mandibular midline deviation with a risk ratio 1.18 (95% CI 0.96, 1.46) (*P* = 0.12; Fig. 9). The heterogeneity was very low (Chi^2^ = 0.02, *df* = 1, *P* = 0.89; *I*^2^ = 0%).

## Second: The RME versus a control group

### Widths of the maxillary and mandibular dental arches

Depending on this systematic review, there was only one RCT that applied RME (Hyrax) to treat FPXB and compared its results with control group. It found a significant increase in the intercanine transversal width of the maxillary arch (mean value: 3.6 mm) compared with the control group (mean value: 1 mm) with a weighted mean difference of 2.60 (95% CI 0.93, 4.27; *P* = 0.002). The upper intermolar width also increased significantly in the Hyrax group (mean value: 5.1 mm) compared with the control group (mean value: 0.8 mm) with a weighted mean difference of 4.30 mm (95% CI 2.05, 6.55; *P* < 0.001). While the measurements of the lower arch did not show any notable differences after the rapid expansion had been applied (26). No meta-analysis can be done.

#### Correction of the mandible midline deviation

After the rapid expansion by Hyrax, the midline deviation decreased about 1.6 mm according to 1 RCT [44]**.** Whereas, the deviation increased in the untreated control group about 0.2 mm. The mean difference between groups was significant (WMD = − 1.80; 95% CI − 2.56, − 1.04; *P* < 0.001).

#### The strength of the evidence in the collected data

According to GRADE analysis and for the first set of comparisons between the QH and the EP, the level of evidence was moderate for the maxillary intermolar width, success rate, and mandibular midline correction outcomes. The treatment duration outcome had also a moderate level of certainty. For the other dental arches' dimensions and other outcomes, the certainty level ranged from low to very low (Table 5). For all the outcomes in the second set of comparisons between the RME and the control groups, the level of evidence was 'very low' (Table 6). The decline in the strength of the evidence occurred because of the high heterogeneity, risk of bias [42–44], or existence of CCTs [39, 41].

**Table 5:** Summary of findings according to GRADE

Quad-helix compared with expansion plate for treatment of functional posterior cross bite	
**Patient or population**: patients in mixed dentition had functional posterior crossbite **Intervention**: quad-helix **Comparison**: expansion plate	

Outcomes	Anticipated absolute effects* (95% CI)		Relative effect (95% CI)	№ of participants (studies)	Certainty of the evidence (GRADE)	Comments
	Risk with expansion plate	Risk with quad-helix				
Maxillary intermolar width—RCTs		MD 1.25 higher (0.75 higher to 1.75 higher)	–	241 (4 RCTs)	⨁⨁⨁◯ MODERATE^a^	The treatment by quad-helix increased the maxillary intermolar width by 1.25 mm with a statistically significant difference compared with expansion plate
Maxillary intermolar width—CCTs		MD 0.23 higher (0.99 lower to 1.45 higher)	–	72 (2 CCTs)	⨁◯◯◯ VERY LOW^b^^,^^c^^,^^d^	Quad-helix may increase/have little to no effect on maxillary intermolar width but the evidence is very uncertain
Maxillary intercanine width—RCTs		MD 0.42 higher (0.55 lower to 1.39 higher)	–	241 (4 RCTs)	⨁◯◯◯ VERY LOW^a^^,^^c^^,^^e^	The evidence suggests that quad-helix may increase the maxillary intercanine width slightly compared with expansion plate and the difference between both groups is non-significant
Maxillary intercanine width—CCTs		MD 0.45 higher (1.87 lower to 2.77 higher)	–	72 (2 CCTs)	⨁◯◯◯ VERY LOW^b^^,^^c^^,^^e^	The evidence suggests that quad-helix may increase/decrease the maxillary intercanine width slightly compared with expansion plate and the difference between both groups is non-significant
Mandibular intermolar width—RCTs		MD 0.3 lower (1.06 lower to 0.47 higher)	–	131 (3 RCTs)	⨁◯◯◯ VERY LOW^a^^,^^c^^,^^f^	Quad-helix may reduce/have little to no effect on mandibular intermolar width compared with expansion plate but the evidence is very uncertain
mandibular intermolar width—CCTs		MD 0.01 lower (0.28 lower to 0.25 higher)	–	72 (2 CCTs)	⨁◯◯◯ VERY LOW^b^^,^^d^^,^^g^	Quad-helix may reduce/have little to no effect on mandibular intermolar width compared with expansion plate but the evidence is very uncertain
Mandibular intercanine width—RCTs		MD 0.39 lower (0.86 lower to 0.09 higher)	–	131 (3 RCTs)	⨁◯◯◯ VERY LOW^a^^,^^d^^,^^h^	Quad-helix may have little to no effect on mandibular intercanine width compared with expansion plate but the evidence is very uncertain
Mandibular intercanine width—CCTs		MD 0.19 higher (0.13 lower to 0.5 higher)	–	72 (2 CCTs)	⨁◯◯◯ VERY LOW^b^^,^^d^^,^^g^	Quad-helix may have little to no effect on mandibular intercanine width but the evidence is very uncertain
Success rate—RCTs	757 per 1,000	977 per 1000 (757 to 1000)	RR 1.29 (1.00 to 1.66)	206 (3 RCTs)	⨁⨁⨁◯ MODERATE^h^	The evidence suggests that quad-helix likely results in an increase in success rate compared with expansion plate and the difference between both groups is significant
Success rate—CCTs	1000 per 1000	1000 per 1000 (910 to 1000)	RR 1.00 (0.91 to 1.10)	38 (1 CCT)	⨁◯◯◯ VERY LOW^b^^,^^i^	The evidence is very uncertain about the effect of quad-helix compared with expansion plate on success rate
Relapse at 1 year post-treatment	91 per 1000	91 per 1000 (20 to 418)	RR 1.00 (0.22 to 4.60)	66 (1 RCT)	⨁⨁◯◯ LOW^i^	The evidence suggests that quad-helix results in no difference in relapse at 1 year post-treatment compared with expansion plate
Relapse at 3 years post-treatment	0 per 1000	0 per 1000 (0 to 0)	RR 2.29 (0.10 to 52.48)	35 (1 RCT)	⨁◯◯◯ VERY LOW^a^^,^^j^	The evidence is very uncertain about the effect of quad-helix and expansion plate on relapse at 3 years post-treatment
Relapse at 5.6 years	53 per 1000	158 per 1000 (18 to 1,000)	RR 3.00 (0.34 to 26.33)	38 (1 CCT)	⨁◯◯◯ VERY LOW^b^^,^^j^	Quad-helix may have little effect on relapse at 5.6 years compared with expansion plate but the evidence is very uncertain
Treatment duration—RCTs		MD 3.36 lower (4.97 lower to 1.75 lower)	–	206 (3 RCTs)	⨁⨁⨁◯ MODERATE^h^	The evidence suggests that quad-helix probably results in a reduction in treatment duration compared with expansion plate
Treatment duration—CCTs		MD 4.8 lower (7.07 lower to 2.53 lower)	–	38 (1 CCT)	⨁◯◯◯ VERY LOW^b^^,^^g^	Quad-helix may reduce the treatment duration compared with expansion plate but the evidence is very uncertain
Mandibular midline correction	644 per 1000	760 per 1000 (618 to 940)	RR 1.18 (0.96 to 1.46)	122 (2 RCTs)	⨁⨁⨁◯ MODERATE^k^	The evidence suggests that quad-helix may increase the midline correction rate compared with expansion plate but the difference between both groups is statistically non-significant
*The risk in the intervention group (and its 95% confidence interval) is based on the assumed risk in the comparison group and the relative effect of the intervention (and its 95% CI) CI: Confidence interval; MD: Mean difference; RR: Risk ratio						
GRADE Working Group grades of evidence High certainty: We are very confident that the true effect lies close to that of the estimate of the effect Moderate certainty: We are moderately confident in the effect estimate: The true effect is likely to be close to the estimate of the effect, but there is a possibility that it is substantially different Low certainty: Our confidence in the effect estimate is limited: The true effect may be substantially different from the estimate of the effect Very low certainty: We have very little confidence in the effect estimate: The true effect is likely to be substantially different from the estimate of effect						

**Explanations**

^a^Unclear risk of bias for random sequence generation and allocation concealment in one included trial

^b^There was a risk of bias in the assessment of the study endpoint and prospective calculation of the study size

^c^There was a significant heterogeneity between the included studies

^d^The boundaries of the CI are not on the same side of their decision-making threshold

^e^The CI crosses the clinical decision-making threshold for an acceptable estimate of treatment

^f^The recommendation will be altered if the lower versus the upper boundary of the CI represent the true underlying effect

^g^The number of patients who provide data is very low

^h^There a was a heterogeneity between the included studies

^i^The event rates are very low

^j^The event rates are low and CI around relative effects is wide

^k^The event rates are low

^l^The CI around relative effects may be wide

^m^The CI around relative effects is wide

**Table 6:** Summary of findings according to GRADE

RME compared with untreated control group for treatment of functional posterior cross bite	
**Patient or population**: patients in mixed dentition had functional posterior crossbite **Intervention**: RME **Comparison**: untreated control group	

Outcomes	Anticipated absolute effects* (95% CI)		Relative effect (95% CI)	№ of participants (studies)	Certainty of the evidence (GRADE)	Comments
	Risk with untreated control group	Risk with RME				
Maxillary intermolar width		MD 4.3 higher (2.05 higher to 6.55 higher)	–	66 (1 RCT)	⨁◯◯◯ VERY LOW^a^^,^^b^	RME may increase the maxillary intermolar width compared with control group but the evidence is very uncertain
Maxillary intercanine width		MD 2.6 higher (0.93 higher to 4.27 higher)	–	66 (1 RCT)	⨁◯◯◯ VERY LOW^a^^,^^b^	RME may increase the maxillary intercanine width compared with control group but the evidence is very uncertain
Mandibular intermolar width		MD 0 (1.51 lower to 1.51 higher)	–	66 (1 RCT)	⨁◯◯◯ VERY LOW^a^^,^^b^^,^^c^	The evidence is very uncertain about the effect of RME on mandibular intermolar width
Mandibular intercanine width		MD 0.8 higher (0.38 lower to 1.98 higher)	–	66 (1 RCT)	⨁◯◯◯ VERY LOW^a^^,^^b^^,^^d^	The evidence is very uncertain about the effect of RME on mandibular intercanine width
Mandibular midline correction		MD 1.8 lower (2.56 lower to 1.04 lower)	–	66 (1 RCT)	⨁◯◯◯ VERY LOW^a^^,^^b^	RME may reduce the mandibular midline deviation compared with control group but the evidence is very uncertain
*The risk in the intervention group (and its 95% confidence interval) is based on the assumed risk in the comparison group and the relative effect of the intervention (and its 95% CI) CI: Confidence interval; MD: Mean difference						
GRADE Working Group grades of evidence High certainty: We are very confident that the true effect lies close to that of the estimate of the effect Moderate certainty: We are moderately confident in the effect estimate: The true effect is likely to be close to the estimate of the effect, but there is a possibility that it is substantially different Low certainty: Our confidence in the effect estimate is limited: The true effect may be substantially different from the estimate of the effect Very low certainty: We have very little confidence in the effect estimate: The true effect is likely to be substantially different from the estimate of effect						

**Explanations**

^a^High risk due to reporting bias

^b^The number of patients who provide data is low

^c^The boundaries of the CI are not on the same side of their decision-making threshold

^d^he recommendation will be altered if the lower versus the upper boundary of the CI represent the true underlying effect

## Discussion

Although the expansion was effective in the correction of the posterior crossbite in mixed dentition but even after the transversal expansion occurred, the treatment groups did not reach the same average width of the maxilla as it was in the control group [41, 43]. According to this systematic review, it appears there is a moderate level of certainty that the treatment by the quad-helix appliance increased the maxillary intermolar width more than the expansion plate. Furthermore, Petern et al. 2008 found out that the intermolar width was greater in the QH group, whereas the intercanine width was greater in the EP group which might be explained by the fact that the arm of the quad-helix did not touch the canines before a certain amount of expansion had taken place in the molar and deciduous molar region [42]. However, the maxillary intercanine width did not differ significantly between both groups. For the mandibular intermolar and intercanine width outcomes, the level of evidence is very low. It seems that both of quad-helix and removable plates resulted in a little to no expansion in mandibular width with no clinical implication. More studies are needed to assess the spontaneous changes that occurred in mandibular arch dimension due to maxillary expansion.

The success rate of correcting the FPXB was judged after the completion of expansion. Depending on previous research work in this field [6, 42, 45]; if the crossbite had not been corrected after 12 months of active expansion, the case would have been considered unsuccessful. According to the three pooled RCTs, the success rate with the quad-helix was higher than the removable plate with a risk ratio of 1.29, and the difference was significant with a moderate level of certainty [6, 42, 45]. This result may be explained by the fact that the successful treatment with removable expansion plates can be achieved only when the patients are cooperative. It was stated that many children in the included trials did not show good cooperation. Thus, the obtained results may not have expressed the actual efficacy of the expansion plates if worn full-time.

Regarding the relapse after slow expansion, three studies evaluated the percentage of relapse with a follow-up period from 1 to five and a half years approximately [6, 41, 43]. However, some studies did not mention any relapse, which could be a result of the short observation period after retention, except for Boysen et al. who did not notice any relapse despite the two-year observation period. The relapse rate in the QH and the EP groups ranged from 5 to 24% [6, 41, 43]. Godoy et al. evaluated the relapse at one-year post-treatment (6 months post-retention) and found no difference between the QH and EP groups (about 9% of patients had a relapse in each group)[6], but the evidence for this outcome is low.

In contrast, Petren et al. 2011 assessed the relapse at three years post-treatment (i.e., 2.5 years nearly post-retention) and found that the relapse rate did not differ significantly [43]. However, according to one CCT by Bjerklin et al., the observation period was 5.6 years (including 3–5 months for retention). It revealed that the relapse in the QH group was three times higher than that in the EP group, however, that difference was non-significant. Although the relapse rate was assessed by all studies and the observed differences were statistically insignificant, relapse rate was remarkably greater in the quad-helix group compared to expansion plate group (as in the Bjerklin et al. study). The insignificant finding may be attributed to the small sample size in this study which affected its statistical power. Relapse following expansion can be explained by the more buccal tipping of the maxillary first permanent molars in the QH group when compared to the EP group. This tipping movement caused by QH is more susceptible to relapse. The evidence is very low; therefore, there is a need for more studies assessing the relapse in the short- and long-term to get a better evidence.

Concerning spontaneous correction of the crossbite, there were not enough studies. According to Petern et al. 2008 spontaneous correction in the mixed dentition did not occur, and the crossbite correction with posterior composite onlay in the mixed dentition was not effective [42]. All of the studies that compared QH and EP showed the same result in which the shorter treatment period was observed in QH groups [6, 39, 41, 42]. The quad-helix was effective at reducing the treatment duration by an average of 3.36 months in this meta-analysis, and the level of certainty for this finding is moderate. This result can be explained by the fact that expansion by the removable plates depends on the patient's compliance. The expansion protocol of the expansion plates also played a role in lengthening the treatment (one-quarter of a full turn each week).

The correction of midline deviation in both EP and QH groups with rates varied from 59 to 93% [40, 42, 43]. Petren et al. 2008 found that the lower midline correction percentage was higher in the QH group than the EP group [42]. According to Sollenius et al. 2020, the percentage of correction was 70% in the QH group and 59% in the EP group [46]. Depending on the performed meta-analysis, there was a non-significant difference between the QH and EP patients, and the evidence is moderate. The treatment with RME followed by a U-bow activator effectively reduced the midline deviation from 2 to 0.5 mm [44], but the evidence is very low and more studies are needed.

## Conclusions

### Implications for practice

There is a weak to moderate evidence that the treatment of FPXB by quad-helix increased the maxillary intermolar width and the success rate and decreased the treatment duration compared to removable expansion plate. However, the relapse percentage was greater in quad-helix groups. There is moderate evidence that the mandibular midline correction rate did not differ significantly between the quad-helix and the expansion plate. The RME using the Hyrax appliance corrected FPXB successfully; however, the strength of evidence in this regard is very low.

### Implications for research

As the quality of evidence ranged between very low to moderate for all the included variables, we confirm the need for more well-conducted RCTs to assess the different appliances used for slow and rapid maxillary expansion in the early treatment of FPXB.

## Supplementary Information

**Additional file 1.** List of the excluded studies and reasons beyond exclusion

**Additional file 2.** Methodological quality of the selected studies according to Cochrane risk of bias tool for randomized controlled trial (ROB1).

## Data Availability

The datasets used and/or analyzed during the current study are available from the corresponding author on reasonable request.

## Abbreviations

FPXB: Functional posterior crossbite
CCTs: Controlled clinical trials
RCTs: Randomized controlled trials
PRISMA: Preferred reporting items for systematic reviews and meta-analyses
MINORS: Methodological index for non-randomized studies
GRADE: Grading of recommendations, assessment, development, and evaluation
MDs: Mean differences
RR: Risk ratio
QH: Quad-helix
EP: Expansion plate
RME: Rapid maxillary expansion
SME: Slow maxillary expansion
