# Supplementary material for: The effectiveness of the early orthodontic correction of functional unilateral posterior crossbite in the mixed dentition period: a systematic review and meta-analysis
Source: Prog Orthod. 2022 Feb 14;23:5. doi: 10.1186/s40510-022-00398-4 (PMC8841381; doi:10.1186/s40510-022-00398-4)
Supplement: Supplementary file 1 — Additional file 1. List of the excluded studies and reasons beyond exclusion [file 40510_2022_398_MOESM1_ESM.docx]

| **Supplementary Table 1: List of the excluded studies and reasons beyond exclusion** | | | |
| --- | --- | --- | --- |
| **Authors/year** | **Publication journal** | **Study title** | **Reason for exclusion** |
| Piancino MG, Cordero-Ricardo M, Cannavale R, Vallelonga T, Garagiola U, Merlo A. 2017 | Angle Orthod | Improvement of masticatory kinematic parameters after correction of unilateral posterior crossbite: Reasons for functional retention. | This study evaluated the reverse-sequencing chewing cycle which is not one of the main outcomes of this systematic review. |
| Galbiati G, Maspero C, Giannini L, Tagliatesta C, Farronato G. 2016 | Minerva Stomatol | Functional evaluation in young patients undergoing orthopedical interceptive treatment. | This study examined the activity of jaw muscles which is not one of the main outcomes of this systematic review. |
| Ugolini A, Doldo T, Ghislanzoni LT, Mapelli A, Giorgetti R, Sforza C. 2016 | Prog Orthod | Rapid palatal expansion effects on mandibular transverse dimensions in unilateral posterior crossbite patients: a three-dimensional digital imaging study | The sample consisted of unilateral posterior crossbite patients without any indication of the presence of a mandibular shift. |
| Weyrich C, Noss M, Lisson JA. 2010 | J Orofac Orthop | Comparison of a modified RME appliance with other appliances for transverse maxillary expansion | The sample consisted of unilateral posterior crossbite patients without any indication of the presence of a mandibular shift. |
| Cozzani M, Rosa M, Cozzani P, Siciliani G. 2003 | Prog Orthod | Deciduous dentition-anchored rapid maxillary expansion in crossbite and non-crossbite mixed dentition patients: reaction of the permanent first molar | The sample consisted of posterior crossbite and non-crossbite patients without any indication of the presence of a mandibular shift. |
| Lippold C, Moiseenko T, Drerup B, Schilgen M, Vegh A, Danesh G. 2012 | BMC Musculoskelet Disord | Spine deviations and orthodontic treatment of asymmetric malocclusions in children | This study assessed the effect of early orthodontic treatment for unilateral posterior cross bite on spine deviations which is not one of the main outcomes of this systematic review. |
| Illipronti-Filho E, Fantini SM, Chilvarquer I. 2015 | Braz Oral Res | Evaluation of mandibular condyles in children with unilateral posterior crossbite | The sample consisted of unilateral posterior crossbite patients without any indication of the presence of a mandibular shift. |
| Ramoglu SI, Sari Z. 2010 | Eur J Orthod | Maxillary expansion in the mixed dentition: rapid or semi-rapid? | The sample consisted of bilateral crossbite patients. |
| Tonni I, Iannazzi A, Piancino MG, Costantinides F, Dalessandri D, Paganelli C. 2017 | Eur J Orthod | Asymmetric molars' mesial rotation and mesialization in unilateral functional posterior crossbite and implications for interceptive treatment in the mixed dentition | This study analysed mesial rotation and mesialization of upper first molars which is not one of the main outcomes of this systematic review. |
| Adly MS, Adly AS. 2020 | J Telemed Telecare | Assessment of early orthodontic treatment on functional shifts by telemonitoring mandibular movements using a smart phone | This study assessed the effect of orthodontic treatment on anterior or lateral functional shift by telemonitoring mandibular movements using smart phone which is not one of the main outcomes of this systematic review. |
| Sandikcioglu M, Hazar S. 1997 | Am J Orthod  Dentofacial Orthop | Skeletal and dental changes after maxillary expansion in the mixed dentition | The sample consisted of unilateral and bilateral posterior crossbite patients without any indication of the presence of a mandibular shift. |
