# Supplementary material for: The effectiveness of the early orthodontic correction of functional unilateral posterior crossbite in the mixed dentition period: a systematic review and meta-analysis
Source: Prog Orthod. 2022 Feb 14;23:5. doi: 10.1186/s40510-022-00398-4 (PMC8841381; doi:10.1186/s40510-022-00398-4)
Supplement: Supplementary file 2 — Additional file 2. Methodological quality of the selected studies according to Cochrane risk of bias tool for randomized controlled trial (ROB1). [file 40510_2022_398_MOESM2_ESM.docx]

| **Supplementary Table 2: Methodological quality of the selected studies according to Cochrane risk of bias tool for randomized controlled trial (ROB1)** | | | | | | | |
| --- | --- | --- | --- | --- | --- | --- | --- |
| Study ID | Random sequence generation | Allocation concealment | Blinding of participants and personnel | Blinding of outcome assessment | Incomplete outcome data | Selective reporting | Other bias |
| Godoy et al., 2011 | Authors’ judgment :  Low risk | Authors’ judgment :  Low risk | Authors’ judgment :  Low risk | Authors’ judgment :  Low risk | Authors’ judgment :  Low risk | Authors’ judgment :  low risk | Authors’ judgment :  Low risk |
|  | Support for judgment :  Quote: “For randomization, numbers were randomly drawn from a plastic bag. Each child received a number from 1 to 99” | Support for judgment :  The randomization method applied would not allow the involved individuals to know their allocation. | Support for judgment :  Blinding of patients and orthodontists could not be applied, and the outcomes are not likely to be influenced by lack of blinding. | Support for judgment :  Quote: “The investigator  was unaware of the type of appliance used by the patient and the length of treatment” | Support for judgment :  Quote:“ Dropouts and treatments not completed  within 12 months were classified as unsuccessful” | Support for judgment :  The measurements of outcomes described in the methods section were all reported. | Support for judgment :  No other sources of bias identified. |
| Lippold et al., 2013 | Authors’ judgment :  Low risk | Authors’ judgment :  Unclear risk | Authors’ judgment :  Low risk | Authors’ judgment :  Unclear risk | Authors’ judgment :  Low risk | Authors’ judgment :  High risk | Authors’ judgment :  Low risk |
|  | Support for judgment :  Quote: “Block randomization with a block length of 20 and an allocation ratio of 1:1” | Support for judgment :  The allocation concealment was not mentioned. | Support for judgment :  Blinding of patients and orthodontists could not be applied, and the outcomes are not likely to be influenced by lack of blinding | Support for judgment :  Although the outcomes assessors could have been blinded, the blinding of outcomes assessment was not mentioned. | Support for judgment :  Numbers of drop-outs and reasons were clearly described in a study workflow (Figure 1), and were sufficiently similar in each  Group. | Support for judgment  No SD for mean change from baseline reported. | Support for judgment :  No other sources of bias identified. |
| Petren et al., 2008 | Authors’ judgment :  Low risk | Authors’ judgment :  Low risk | Authors’ judgment :  Low risk | Authors’ judgment :  Low risk | Authors’ judgment :  Low risk | Authors’ judgment :  Low risk | Authors’ judgment :  Low risk |
|  | Support for judgment :  Quote: “The subjects were randomized as follows: 4 opaque envelopes were prepared with 20 sealed notes in each (5 notes for each group). Thus, for every new patient in the study, a note was extracted from the first envelope. When the envelope was empty,the second envelope was opened, and the  20 new notes were extracted as patients were recruited to the study. This procedure  was then repeated 2 more times” | Support for judgment :  Quote: “The envelope was in the care of 1 investigator...who was contacted and randomly extracted a note and informed the dentist which treatment strategy to use” | Support for judgment :  Blinding of patients and orthodontists could not be applied, and the outcomes are not likely to be influenced by lack of blinding. | Support for judgment :  Quote: “Measurements were blinded; the  examiner was unaware of which treatment  the patients had received or which models  were taken at T0 and T1” | Support for judgment :  Quote: “Data on all patients were analyzed on an intention-to-treat (ITT) basis... all patients, successful or not, were included in  the final analysis” and “all patients finished the trial (Fig 5)” | Support for judgment :  The measurements of outcomes described in the methods section were all reported. | Support for judgment :  No other sources of bias identified. |
| Petren et al., 2011 | Authors’ judgment :  Unclear risk | Authors’ judgment :  Unclear risk | Authors’ judgment :  Low risk | Authors’ judgment :  Low risk | Authors’ judgment :  Low risk | Authors’ judgment :  Low risk | Authors’ judgment :  Low risk |
|  | Support for judgment :  Quote: ''Most (n 5 30) of the crossbite patients were recruited from an RCT study''.  It appears that 30 patients of the sample were recruited from Petren et al. 2008 study. However, the randomization method of the rest of patients was not mentioned. | Support for judgment :  The allocation concealment was not mentioned. | Support for judgment :  Blinding of patients and orthodontists could not be applied, and the outcomes are not likely to be influenced by lack of blinding. | Support for judgment :  Quote: ''Assessment of transverse occlusion (crossbite correction) and all study cast measurements were blinded; ie, the examiner was unaware of the group to which the patient belonged. Furthermore, the T0, T1, and T2 casts were randomized for measurement''. | Support for judgment :  Numbers of drop-outs and reasons were clearly described in a study workflow (Figure 1). | Support for judgment :  The measurements of outcomes described in the methods section were all reported. | Support for judgment :  No other sources of bias identified. |
| Sollenius et al., 2019 | Authors’ judgment :  Low risk | Authors’ judgment :  Low risk | Authors’ judgment :  Low risk | Authors’ judgment :  Low risk | Authors’ judgment :  Low risk | Authors’ judgment :  Low risk | Authors’ judgment :  Low risk |
|  | Support for judgment :  Quote: ''The randomization was prepared and carried out by an independent  person not involved in the study and the randomization used blocks  of 25 (5 + 5 + 5 + 5 + 5). Thus, the orthodontists carried boxes of already prepared envelopes. Six opaque envelopes were prepared with 25 sealed notes in each (5 notes for each group), and for every new patient in the study, a note was extracted from the first envelope. When the envelope was empty, the second envelope was opened, and  the 25 new notes were extracted as the patients were recruited to the trial. This procedure was then repeated five more times''. | Support for judgment :  The randomization method applied would not allow the involved individuals to know their allocation. | Support for judgment :  Quote: ''Children and trial personnel could not be blinded due to the character of treatment''. | Support for judgment :  Quote: ''but blinding was performed of the outcome evaluator and the person who analyzed the data. Hence, the evaluator was unaware of the group to which the child had been allocated  or whether the data were from baseline, follow-up, or after treatment''. | Support for judgment :  Numbers of drop-outs and reasons were clearly described in a study workflow (Figure 2). | Support for judgment :  The measurements of outcomes described in the methods section were all reported. | Support for judgment :  No other sources of bias identified. |
| Sollenius et al., 2020 | Authors’ judgment :  Low risk | Authors’ judgment :  Low risk | Authors’ judgment :  Low risk | Authors’ judgment :  Low risk | Authors’ judgment :  Low risk | Authors’ judgment :  Low risk | Authors’ judgment :  Low risk |
|  | Support for judgment :  Quote: ''The randomization procedures were prepared and carried out by an independent person not involved in the study and the randomization used blocks of 20. Thus, the orthodontists carried boxes of already prepared envelopes. Six opaque envelopes were prepared with 20 sealed notes in each (5 notes for each group) and for every new patient in the study, a note was extracted from the first envelope''.  When the envelope was empty, the second envelope was opened,  and the 20 new notes were extracted as the patients were recruited  to the trial. This procedure was then repeated 4 more times. | Support for judgment :  The randomization method applied would not allow the involved individuals to know their allocation. | Support for judgment :  Quote: ''Patients and trial staff could not be blinded because of the character of treatment''. | Support for judgment :  Quote: ''but blinding was accomplished of the outcome assessor;  thus, the assessor was unaware of the group to which the patient belonged or whether the data were for before or after treatment''. | Support for judgment :  Numbers of drop-outs and reasons were clearly described in a study workflow (Figure 3). | Support for judgment :  The measurements of outcomes described in the methods section were all reported. | Support for judgment :  No other sources of bias identified. |
